# Better sleep is associated with higher academic performance from an actigraphy-based analysis of sleep consistency and grades in college students

**DOI:** 10.1038/s41598-025-33775-0

**Published:** 2025-12-26

**Authors:** Chen-Ta Lin, Sheng-Fu Liang, Fu-Zen Shaw

**Affiliations:** 1 Department of Computer Science and Information Engineering, National Cheng Kung University, No. 1, University Rd., East Dist, Tainan, 70101 Taiwan (R.O.C.); 2 Department of Psychology, National Cheng Kung University, No. 1, University Rd., East Dist, Tainan, 70101 Taiwan (R.O.C.)

**Keywords:** Health care, Cognitive neuroscience, Learning and memory

## Abstract

Academic performance is influenced by sleep; however, the relationship between academic outcomes and subjective sleep measures remains controversial. This study aimed to evaluate the association between sleep parameters and academic performance using objective actigraphy measurements alongside subjective assessments. University students (*n* = 33) enrolled in a sleep psychology course participated in the study and completed all assessments. Participants completed three subjective questionnaires and 14 consecutive days of actigraphy recordings. They were retrospectively divided into high- and low-performing groups based on the median final exam score. Four objective sleep parameters derived from actigraphy were analyzed: sleep efficiency (SE), sleep onset latency (SOL), wake after sleep onset, and total sleep time (TST). Additionally, the mean absolute deviation (MAD) was introduced to assess night-to-night variability in these parameters. Results showed no significant group differences or correlations between subjective sleep measures and academic performance. In contrast, students with higher exam scores exhibited significantly greater SE, shorter SOL, and lower MADs for SE, SOL, and TST. Final exam scores were significantly associated with SE, SOL, and the MADs of SE, SOL, and TST. These associations were especially pronounced on weekdays, where exam scores correlated with SE, SOL, and the MADs of SE and SOL. Our findings highlight the importance of consistent, high-quality sleep during weekdays for academic success. Furthermore, this study contributes novel insights into the predictive value of night-to-night variability (MAD) in sleep parameters for academic performance. Overall, the results underscore the critical role of stable and efficient weekday sleep in supporting positive academic outcomes.

**Supplementary Information:**

The online version contains supplementary material available at 10.1038/s41598-025-33775-0.

## Introduction

Humans spend approximately one-third of their lives asleep. The role of sleep in enhancing various cognitive functions has been an interesting topic for over a century. Numerous well-controlled laboratory studies have demonstrated that sufficient sleep increases cognitive functions, whereas sleep deprivation impairs it^1,2^, particularly for learning and memory^3,4^. In recent years, a growing number of studies have moved beyond laboratory settings by utilizing both self-reported measures^5,6^ and objective sleep measures^7^ collected from students in their natural living environments, to further investigate the relationship between sleep and cognitive functioning.

In general, sleep is associated with cognitive performance^8^. Sleep disturbances or sleep deficits caused by sleep deprivation have been associated with reduced cognitive control and diminished attention during class^9,10^. Conversely, a satisfactory nap can significantly alleviate sleepiness and improve cognitive performance^11^. Numerous studies have shown that self-reported sleep parameters (e.g., sleep quality and total sleep time (TST) of sleep diary or Pittsburgh Sleep Quality Index (PSQI)) are either positively associated with academic performance^12–14^ or show no associations^15,16^. These inconsistent findings highlight the ongoing controversy surrounding the use of self-reported sleep measures. In addition to correlational studies, group comparison designs can provide more insight into the causal relationship between sleep and academic performance. However, only a few studies have conducted post hoc group analyses to understand sleep on academic performance using self-reported sleep measures^17,18^. While one study found that students in the high-score group had significantly better subjective sleep quality^18^, another reported no significant group differences^17^. Taken together, subjective sleep parameters of diaries/PSQI may not be good enough to understand the effect of sleep on cognitive function.

The PSQI has been widely used to investigate the relationship between sleep and academic performance^12,16,19^. Of these studies, the PSQI score or TST of the PSQI has shown inconsistent findings regarding their association with academic performance. The PSQI and its components are susceptible to subjective bias and sleep misperceptions, as the questionnaire relies on retrospective self-reporting of sleep over a one-month period^20–22^. Moreover, it does not capture night-to-night variations or distinctions between weekday and weekend sleep patterns. Measuring such variation is important, as many individuals accumulate sleep debt during weekdays and compensate by oversleeping on weekends^23^. Weekly fluctuations in sleep behavior are common and have been shown to affect academic outcomes^24,25^. For instance, adolescents who exhibit greater sleep inconsistency perform worse in school^25^. In addition, self-reported TST is frequently overestimated and often inconsistent when compared with objective TST measured by actigraphy^26,27^ or polysomnography (PSG)^28^. While PSG remains the gold standard for sleep assessment, it is difficult to implement in home settings and incurs high costs for long-term recording. In this study, objective actigraphy was used to continuously track sleep and wakefulness over a two-week period in participants’ daily lives^29,30^.

Academic or work performance has been associated with various subjective sleep behaviors or sleep habits, such as TST^27,31^, regularity of bedtime across days^32^, or sleep quality^33^. Clinically, sleep quality is often quantified using specific parameters such as sleep efficiency (SE), sleep onset latency (SOL), and wake after sleep onset (WASO)^34^. These clinical parameters provide more detailed properties about sleep quality to identify the patterns of sleep disturbance. A study recruiting pharmacy college students found that SE of a diary measure was positively associated with semester performance, but SE of actigraphy had no association with semester scores^27^. SOL of subjective sleep diary showed inconsistent association with academic performance among four semesters^27^. There is no report of WASO’s impact on academic performance. In addition to the four clinical sleep parameters, variability or regularity of a sleep habit or sleep parameter has recently received great attention^30,35^ but has not been systematically evaluated yet. To further understand the relationship between sleep and academic performance, these sleep parameters and their variabilities on academic performance remain to be determined.

Commercial wristbands, e.g., the Fitbit Charge HR (Fitbit Inc.)^30^, Huawei Honor Watch S1 (HUAWEI Ltd.)^36^, and Motionwatch-8 actigraph (CamNtech Ltd)^27^, have been used to assess TST in relation to academic performance. However, these studies provided limited information regarding other sleep parameters or the regularity of sleep and their associations with academic performance. In this study, we used the actigraphy device and the algorithm, validated with polysomnography (PSG)^37^, to calculate three clinical sleep parameters. The actigraphy device has been widely used to measure sleep and daily activity in older adults within community-based studies^29^. In our preliminary analysis, we found that both SE and its mean absolute deviation (MAD) were associated with academic performance^38^. Building on these findings, the present study employed actigraphy recordings alongside subjective questionnaires to examine academic performance in relation to both the average levels and night-to-night variability of four clinical sleep parameters, while also accounting for differences between weekday and weekend. Prior research has highlighted the strong association between sleep regularity and a range of health-related outcomes, underscoring the importance of consistent sleep patterns^30,39,40^. In this study, we introduced a new metric-MAD-to quantify variability in sleep parameters across nights.

Our analysis examined the relationship between final exam scores and sleep parameters alongside post hoc experimental group analyses to evaluate the impact of the clinical sleep parameters. We hypothesized that final exam scores would be influenced by specific clinical sleep parameters and their variability as measured by the actigraphy.

## Results

This study presented no significant differences between the two groups in terms of age, gender, years of education, body mass index (BMI), Beck Depression Inventory (BDI), or Perceived Stress Scale (PSS) scores. Additionally, no significant differences existed in PSQI or its components, including SOL, TST, and SE (Table 1).

**Table 1:** Demographics and basic characteristics in the two groups.

Variable	LS (*n* = 16)	HS (*n* = 17)	*p* value
Gender (male/female)	7/9	2/15	0.057
Education (year)	14.4 ± 0.7	14.7 ± 0.9	0.218
Age (year)	20.8 ± 1.0	21.1 ± 1.0	0.382
BMI	22.7 ± 4.7	21.8 ± 3.5	0.449
BDI	12.6 ± 10.9	8.4 ± 8.6	0.219
PSS	19.1 ± 8.4	16.1 ± 8.1	0.348
PSQI	6.4 ± 3.2	5.9 ± 2.8	0.744
PSQI_SE(%)	95.1 ± 9.4	88.1 ± 24.1	0.251
PSQI_ SOL(min)	24.8 ± 19.0	15.7 ± 14.1	0.176
PSQI_TST(hour)	7.0 ± 1.8	6.9 ± 1.8	0.767

*BDI* Beck depression inventory, *BMI* body mass index, *HS* high score (score ≥ median), *LS* low score (score < median), *PSQI* Pittsburgh sleep quality index, *PSQI_SE* sleep efficiency in PSQI, *PSQI_SOL* sleep onset latency in PSQI, *PSQI_TST* total sleep time in PSQI, *PSS* perceived stress scale.

### Association between objective sleep parameters and academic performance

**Fig. 1:**
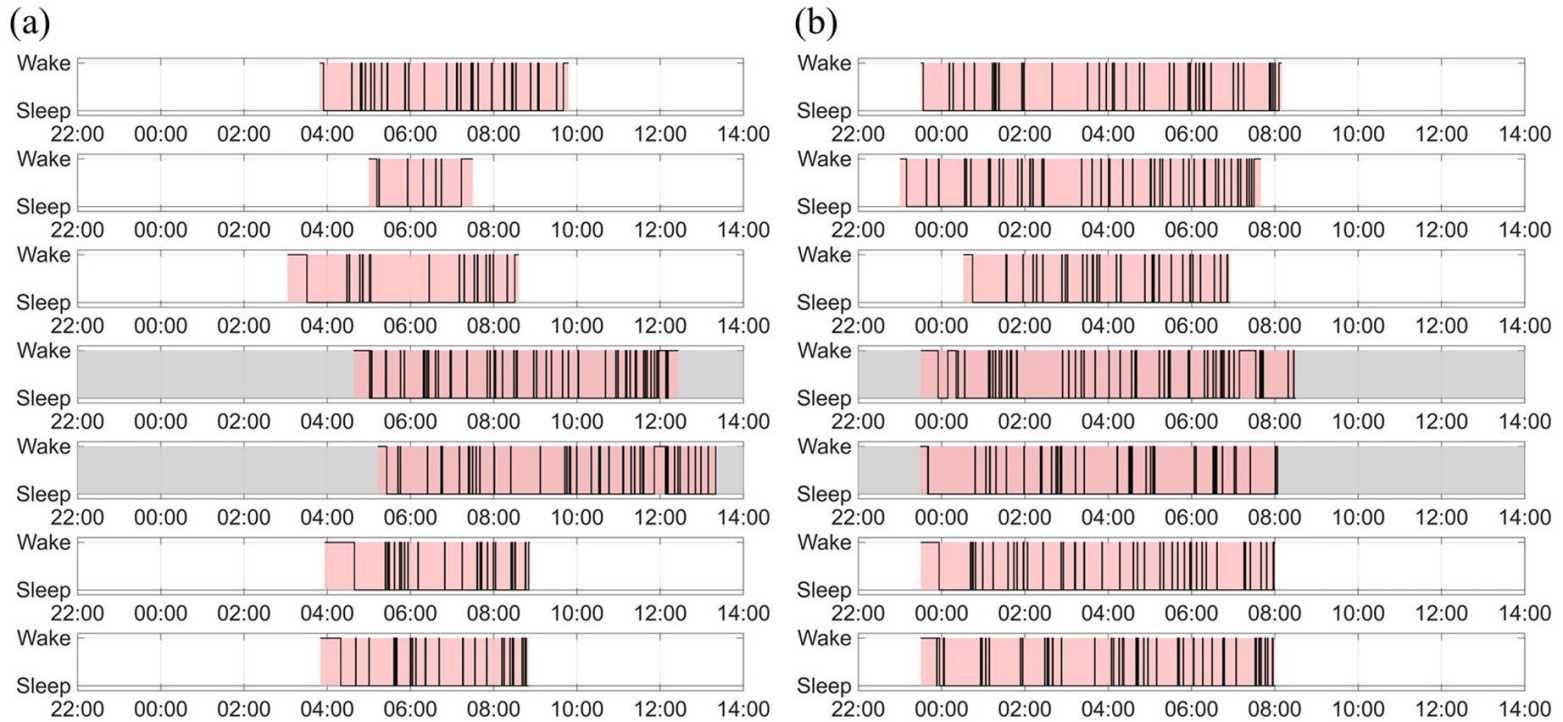
Seven-day sleep–wake cycles for two representative students from the LS group (**a**) and the HS group (**b**). The gray background indicates the weekend period, while the pink background represents time spent in bed. Sleep data were derived using an actigraphy algorithm with 30-second epochs during time in bed.

Representative examples of seven-day sleep–wake cycles are presented in Fig. 1, including one student from the LS group (Fig. 1a) and one from the HS group (Fig. 1b). The LS student exhibited short sleep durations on weekdays, longer sleep durations on the weekend, and considerable variability over the seven-day period. In contrast, the HS student demonstrated a more consistent sleep pattern across the week compared to the LS student.

We first examined four actigraphy-based sleep parameters between the two groups (Table 2). SE was significantly higher in the HS group than in the LS group, with large effect size (*r* = 0.68). SOL was significantly lower in the HS group than in the LS group, also with large effect size (*r* = 0.58). No significant differences were found between the groups for WASO or TST.

**Table 2:** Comparison of four objective sleep parameters and their mads between low score and high score groups.

Variable	LS	HS	*p* value
SE (%)	83.8 ± 3.1	88.8 ± 2.4	**< 0.001**
SOL (min)	26.9 ± 12.1	15.3 ± 4.5	**< 0.001**
WASO (min)	38.3 ± 17.1	29.8 ± 10.4	0.177
TST (hour)	6.0 ± 1.1	6.3 ± 0.8	0.301
MAD			
SE (%)	6.1 ± 3.3	3.4 ± 1.2	**< 0.001**
SOL (min)	16.4 ± 8.3	10.5 ± 6.4	**0.020**
WASO (min)	15.4 ± 7.2	12.6 ± 6.2	0.228
TST (hour)	1.3 ± 0.4	0.9 ± 0.4	**0.010**

*HS* high score (score ≥ median), *LS* low score (score < median), *SE* sleep efficiency, *MAD* mean absolute deviation, *SOL* sleep onset latency, *TST* total sleep time, *WASO* wake after sleep onset. Significant values (p<0.05) are in bold.

Furthermore, the present study considered the night-to-night variability of all sleep parameters between the two groups (Table 2). MAD values of most sleep parameters, including SE (large effect size, *r* = 0.6), SOL (medium effect size, *r* = 0.41), and TST (large effect size, d = 0.96) in the HS group were significantly lower than those of the LS group. There was no difference in the WASO’s MAD between the two groups.

**Fig. 2:**
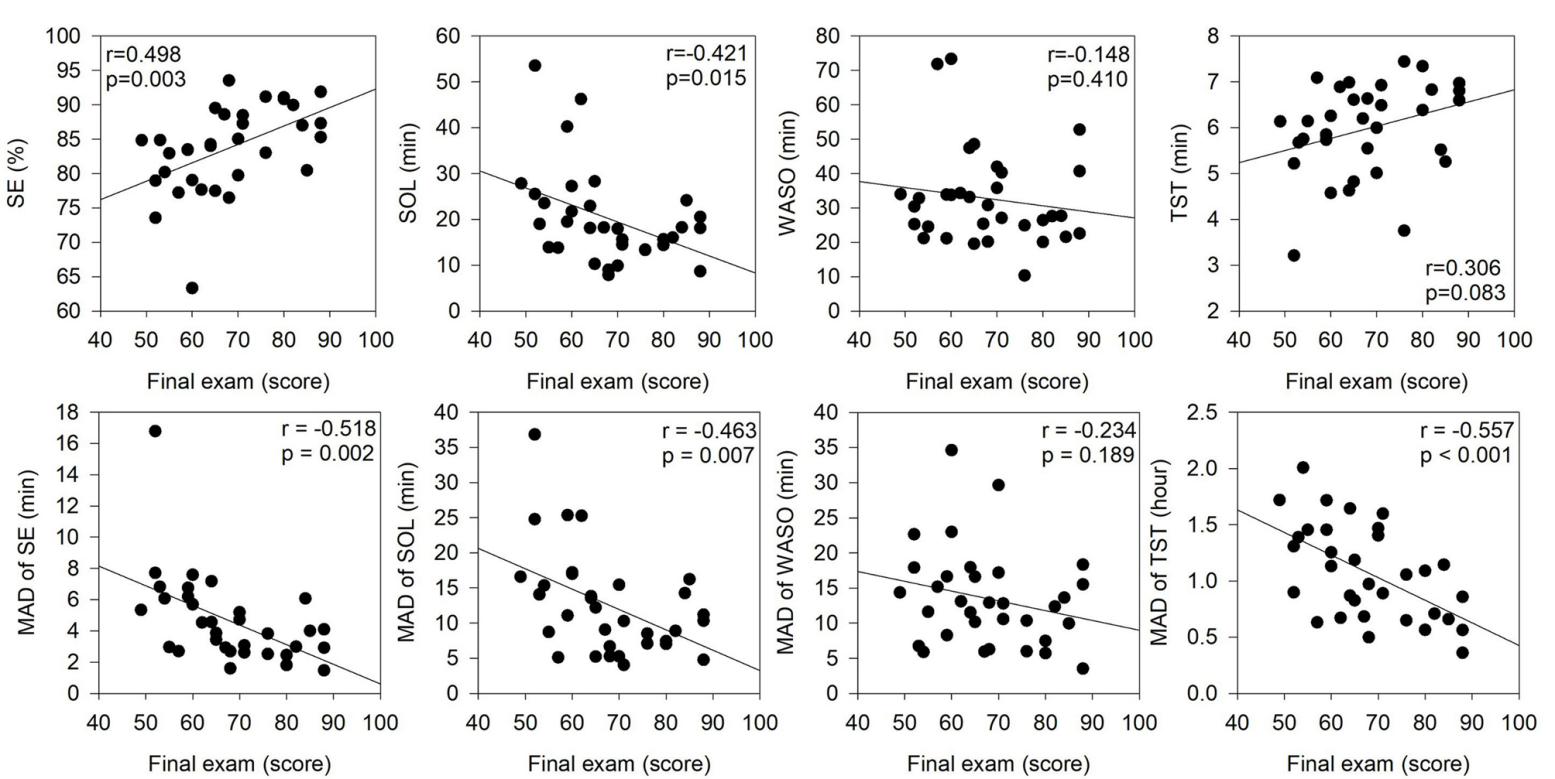
Correlation between the final exam score and sleep parameters.

Furthermore, the current study examined the correlations between final exam scores and various sleep parameters, as illustrated in Fig. 2. A significant positive correlation was found between the final exam score and SE. A significant negative correlation was observed between the final exam score and SOL. No significant correlation was found between the final exam score and WASO or TST. Regarding night-to-night variability, significant negative correlations were observed between the final exam score and the SE’s MAD, SOL’s MAD, and TST’s MAD. However, no significant correlation was found between the final exam score and the MAD of WASO.

### Comparing the effects of weekday and weekend sleep on academic performance

The current study further explored the effects of weekdays and weekends on sleep performance in the two groups (Table 3). The HS group exhibited higher SE, shorter SOL, and lower WASO than the LS group under both weekday and weekend conditions. Correspondingly, SE, SOL, and WASO showed significant main effects of group only with medium to large effect size (Cohen’s f values of 0.29–0.49), with no significant effects of time or group-by-time interaction. In contrast, TST was significantly shorter on weekdays than on weekends in both groups. A significant main effect of time with medium effect size (Cohen’s f = 0.29) was observed for TST, but no group or interaction effects were found. Moreover, the present study explored the correlation between final exam scores and four sleep parameters under weekday and weekend conditions (Table 4). The final exam score exhibited a significant positive correlation with weekday SE but not with weekend SE. A significant negative correlation was observed between final exam scores and weekday SOL, while no such relationship was found for weekend SOL. No significant correlations were found between final exam scores and WASO or TST under either weekday or weekend conditions.

**Table 3:** Comparison of four sleep parameters and their mads in between low score and high score groups under weekday/weekend situations.

	Weekday		Weekend		Factor		
	LS	HS	LS	HS	Group	Time	Grouptime
SE (%)	83.3 ± 3.4	89.0 ± 2.5	85.0 ± 4.8	87.3 ± 6.0	13.58 (**< 0.001**)	0.002 (0.968)	2.42 (0.125)
SOL (min)	28.4 ± 15.3	14.3 ± 3.3	23.1 ± 11.2	17.4 ± 8.7	14.84 (**< 0.001)**	0.17 (0.683)	2.67 (0.107)
WASO (min)	35.8 ± 15.4	29.2 ± 11.5	44.4 ± 28.0	31.1 ± 12.2	5.09 (**0.028)**	1.44 (0.235)	0.57 (0.451)
TST (hour)	5.7 ± 1.2	6.3 ± 0.8	6.7 ± 1.1	6.5 ± 1.1	0.40 (0.528)	5.34 (**0.024)**	2.29 (0.135)
MAD							
SE (%)	6.0 ± 2.8	3.1 ± 1.2	5.3 ± 5.2	3.6 ± 3.9	7.04 (**0.010**)	0.02 (0.894)	0.41 (0.523)
SOL (min)	15.8 ± 8.7	7.7 ± 2.8	14.3 ± 11.6	9.6 ± 5.3	11.39 (**0.001**)	0.01 (0.920)	0.77 (0.383)
WASO (min)	13.9 ± 5.2	10.9 ± 8.4	13.8 ± 11.7	11.2 ± 7.1	1.8 (0.186)	0.001 (0.971)	0.01 (0.919)
TST (hour)	1.1 ± 0.38	1.0 ± 0.5	1.3 ± 0.7	0.9 ± 0.5	6.9 (**0.012**)	0.2 (0.654)	0.96 (0.331)

^a^F value (p value). Significant values (p<0.05) are in bold.

**Table 4:** Correlations between the final exam score and four sleep parameters and their MADs of weekday or weekend situations.

	Weekday				Weekend			
	SE	SOL	WASO	TST	SE	SOL	WASO	TST
Final exam	0.635 (**<0.001**)^a^	-0.463 (**0.006**)	-0.183 (0.307)	0.303 (0.087)	0.051 (0.78)	-0.179 (0.318)	-0.136 (0.45)	-0.036 (0.841)

	M_SE^b^	M_SOL	M_WASO	M_TST	M_SE	M_SOL	M_WASO	M_TST
Final exam	-0.561 (**<0.001**)	-0.469 (**0.006**)	-0.269 (0.130)	-0.433 (**0.012**)	-0.175 (0.329)	-0.265 (0.137)	-0.020 (0.913)	-0.383 (**0.028**)

^a^r value(p value), ^b^MAD value of the parameters. Significant values (p<0.05) are in bold.

Table 3 also shows the variability of sleep parameters under weekday and weekend conditions for the two groups. The MADs of SE, SOL, and TST were lower in the HS group than in the LS group under both weekday and weekend periods. Thus, there were significant differences with medium to large effect size (Cohen’s f values of 0.33–0.43) in the Group factor in MAD values of the SE, SOL, and TST exclusively under the weekday/weekend states. Furthermore, final exam scores were significantly negatively correlated with the weekday MADs of SE, SOL, and TST, as well as with the weekend MAD of TST (Table 4).

## Discussion

This study recruited university students in a sleep-related course and used the final exam score of the course to categorize two groups: LS (score < median) and HS (score≥median). All subjective measures, e.g., BDI, PSS, and PSQI, did not differ significantly between the two groups. In contrast, the HS group exhibited significantly higher SE, and shorter SOL than the LS group. Moreover, the HS group showed significantly lower MAD values in SE, SOL, and TST compared with the LS group. The final exam score exhibited significant correlations with SE, SOL, and MADs of SE, SOL, and TST. In the analysis of weekday and weekend sleep performance, SE, SOL, and WASO showed significant main effects of group, while TST showed a significant main effect of time. Additionally, MAD values of the SE, SOL, and TST exhibited significant main effects in the Group factor. Furthermore, final exam scores were significantly correlated with weekday SE and SOL, weekday MADs of SE, SOL, and TST, and weekend TST. Taken together, our results suggest that a higher achievement (final exam score here) is greatly contributed by better SE, shorter SOL, and greater sleep regularity on weekdays.

This study did not observe significant differences in subjective PSQI score and its components (e.g., SE, SOL, and TST in Table 1) between LS and HS groups. In contrast, objective actigraphy data revealed significantly higher SE and shorter SOL in the HS group compared to the LS group (Table 2). Prior studies often used PSQI to assess students’ sleep quality^12,15,16^. Among these studies, they appeared largely controversial within different subjective sleep reports/parameters and the correlations between sleep parameters and achievement/learning behaviors. Several reasons may account for these discrepancies in the two measures. First, PSQI can only assess sleep quality on a monthly scale and cannot provide sleep quality on a night-to-night basis. It raises a low temporal resolution issue compared with actigraphy. Second, PSQI is scored on limited ordinal or Likert-type scales. Third, PSQI results may be affected by interpretation bias and sleep misperception^20–22^. On the other hand, actigraphy provides objective measurements, it has been widely used in sleep research and has demonstrated moderate to high agreement with gold-standard Polysomnography (PSG) in clinical validation studies^37,41,42^. Actigraphy’s multi-day recording capability helps mitigate potential biases from single-night PSG recordings (e.g., the first-night effect^43^ and from month-level averaging in PSQI. Furthermore, actigraphy can provide high-sensitivity sleep patterns by night-to-night or weekday-weekend resolution. These advantages may explain why significant differences were observed in most actigraphy-derived sleep parameters. Our findings suggest that a relatively high-resolution and precise sleep measure tool (actigraphy device here) can be used and provide more valuable information in the sleep study.

In this study, the HS group exhibited significantly higher SE and shorter SOL compared to the LS group. Moreover, the HS group showed significantly lower MAD values in SE, SOL, and TST compared with the LS group. The MAD values have been demonstrated to offer simplicity, intuitive understanding, and robustness against the distortion caused by extreme values^44^. Here, the MAD values were used to reflect the inter-daily variability. Our results indicate high night-to-night consistency of sleep behavior occurring in the HS group. In general, participants reported sleep disturbance problems or not according to the objective PSQI measure. Few participants in this study reported poor sleep quality (Table 1). In contrast, we identified and characterized their patterns and susceptibilities of sleep disturbances (low efficiency during sleep, difficulty falling asleep with long SOL, inter-daily inconsistency of falling asleep and sleep duration) in terms of clinical sleep parameters and their MADs by actigraphy. Taken together, our study suggests at least 2 valuable clinical sleep parameters (SE & SOL) and their MADs to be used in further sleep investigations regarding achievement.

In addition to sleep parameters of night-to-night measures for an entire recording period, this study also concerned applications of these sleep parameters in weekday and weekend considerations. We found significant differences in SE, SOL, WASO, MAD values of SE, SOL, and TST in the Group factor (Table 3). These data also emphasize significant measure abilities of SE, SOL and their MADs on the weekends/weekdays issues. On the other hand, the TSTs of the two groups showed significant differences in the Time factor (i.e., long TST on the weekend in both groups) exclusively. The phenomenon of longer TST on weekends has been reported in many previous studies^24,25^, which is associated with sleep debt on weekdays^23^ or social jet-lag^39,40,45^. Additionally, TST’s MADs of the two groups expressed no difference in weekday/weekend manipulations. Our results indicate that the two groups exhibit similar sleep behavior by TST variance between weekdays and weekends. According to these results, we provide more evidence to strengthen applications of 2 valuable clinical sleep parameters and their MADs in sleep research for different time periods measures regarding achievement.

Students of this study were recruited from various departments in a single course. There was no difference in all parameters of PSQI regarding the final exam score. By contrast, the final exam score was significantly affected by SE and SOL or MADs of SE, SOL, TST (Table 2), and significant correlations were also expressed with SE, SOL, and their MADs of SE, SOL, TST by actigraphy measures (Fig. 2). These phenomena were partially supported by a previous study that recruited students from a Solid State Chemistry course and used actigraphy measures to observe significant correlations between overall score and TST and its standard deviation within an entire semester^30^. In previous studies that recruited medical students, high and low GPA groups of Iranian medical students expressed no significant difference in sleep quality of PSQI^17^. In contrast, Sudanese medical students with higher GPA scores have a significantly better sleep quality by PSQI^18^. The first reason to explain these controversial findings between our results and previous studies is due to different measures (actigraphy and PSQI), discussed in this section’s second paragraph. The second reason may arise from different achievement measures (single course vs. GPA). GPA reflects a variety of knowledge for overall academic performance, and it largely relies on personal preferences and could be affected by teaching qualities in required and elite courses. Accordingly, GPA measures may produce a relatively great variability to create these controversial phenomena between GPA achievement and sleep quality previously. In our study, we used a single course (i.e., sleep physiology), which is an elite course, to reduce possible individual differences of preference and to reserve teaching quality. Moreover, all participants were from various departments of NCKU and neighboring universities to increase the generalization of this study. Numerous studies in systematic review articles have shown the association between sleep and academic performance^19,46^. Most of them discussed the relationship between TST and academic performance using a diary, PSQI, and/or actigraphy device. Of these studies, they seldom report the relationship between SE/SOL and academic performance simultaneously. In this study, we observed that higher SE and lower SOL are associated with better academic performance and vice versa, but not for TST. Compared with the previous studies with nearly hundreds of participants^30,47^, this study recruited 33 participants, which is a relatively smaller sample size. It might be a reason to get a weakly positive association between TST and academic performance. In addition to understanding the association between academic performance and mean sleep parameters, this study provided advanced information on the association between academic performance and inter-daily variability (i.e., MAD) of SE, SOL, and TST. More consistent night-to-night sleep parameters (i.e., lower MAD values of SE, SOL, and TST in Fig. 2) were associated with better academic performance. Recent studies have also reported a significantly negative relationship between the standard deviation of TST and academic performance in adolescents^48^ and college students^30^. Taken together, classical clinical sleep parameters (SE & SOL) and the variability of SE, SOL, and TST may provide additional information on the association between sleep and academic performance.

In addition to measuring the association between sleep parameters of entire periods and academic performance, we wonder whether the sleep parameters and their consistency of sleep behavior affect academic performance differently on weekdays and weekends. No association was found between sleep parameters on weekends and academic performance (Table 4). Instead, significant correlations occurred between the final exam score and weekdays’ SE/SOL or weekdays’ MAD values of SE, SOL, and TST. In addition to different associations between sleep parameters and academic performance within the entire recording times in previous studies^27,30,36,39^, our findings support great contributions from weekdays rather than weekends. Participants are primarily under continuous learning during weekdays, and they rest or attend extracurricular activities during weekends. Weekdays’ learning may be more directly related to academic learning and performance. It may be a reason to support the importance of weekday sleep measures to explore the association with academic performance.

The present study showed that students with higher academic performance (i.e., higher final exam scores) were accompanied by higher subjective sleep quality (i.e., higher SE), little disturbance to falling asleep (i.e., shorter SOL), and more consistent night-to-night sleep behavior (i.e., lower MAD values of sleep parameters), particularly within the weekday period. Numerous previous studies have indicated sleep being beneficial to memory consolidation after nocturnal sleep^49,50^. Of these studies, immediate post-learning sleep significantly enhances the next-day performance. A previous study showed that pre-sleep learning had significant enhancement after the first night of sleep but did not exhibit a comparable effect on the 4th night of sleep^51^. These data may partially support our significant results of sleep benefits on academic performance occurring weekday sleep parameters, i.e., immediate learning and sleep effect during weekday period. On the other hand, better sleep quality often results in better attention^9^ and better working performance^52^, which plays a crucial role in daytime learning and potentially increases memory consolidation of sleep. These studies may provide an alternative explanation to support our observation of the HS group with high SE and low SOL. Additionally, the circadian variance of sleep patterns plays an important role in the enhancement of several cognitive functions, including attention, learning, and memory^53^. The circadian consistency may be further beneficial to reach a better academic performance. Taken together, our results provide additional evidence on understanding the relationship between academic performance and sleep.

This study has several limitations. First, participants were recruited from a single sleep-related course that included students from various departments. These students may have had a particular interest in the topic of sleep, introducing potential selection bias. To improve the generalizability of future research, participants should be recruited from a broader range of courses and academic backgrounds. Second, the sample in this study consisted predominantly of young female students. Previous research has shown that the menstrual cycle can influence both sleep quality^54^ and academic performance^55^. However, this study did not collect menstrual cycle data during the two-week recording period. Future studies should account for this factor to better understand its potential impact on sleep and academic outcomes. Third, although there were no statistically significant gender differences between the two groups, the p-value approached the threshold for significance. Notably, the HS group included a higher proportion of female participants. While this study reported statistical results using ANOVA, which does not account for confounding variables such as gender, we also conducted analyses using ANCOVA to control for gender as a confounding factor. The majority of the significant sleep metrics reported in Tables 2 and 3 remained significant after this adjustment (Tables S1 & S2 in the supplementary file). However, if future studies find significant gender differences, it will be essential to further account for gender as a potential confounding factor. Fourth, this study observed significantly longer TST on the weekend compared with those of weekdays in both groups. To better understand the potential influence of sleep debt or social jet lag on this weekend sleep extension phenomenon^39,40,45^, future research should include assessments of sleep phase timing. Finally, this study recruited a relatively small sample size. However, most of the significant differences observed in the four sleep metrics and their MADs were associated with medium to large effect sizes. To enhance the interpretability and generalizability of the findings, future studies should consider increasing the sample size.

In summary, our findings indicate that good and consistent sleep quality is significantly associated with better academic performance, as measured by objective actigraphy-based sleep metrics. This study also introduces a novel metric-MAD-to quantify night-to-night variability in sleep and demonstrates its value in assessing the impact of sleep patterns on academic outcomes. Overall, stable and efficient weekday sleep appears to play a crucial role in supporting positive academic performance.

## Methods

### Participants

A total of 82 undergraduate students enrolled in a sleep physiology course at NCKU. They were undergraduate students from NCKU and nearby universities. The course introduced sleep questionnaires, sleep diaries, and actigraphy through lectures and homework assignments. Students were randomly assigned to 18 learned groups, each consisting of 4–5 members. In the course, there was a final group project with the integration of sleep questionnaires, sleep diary, and wearing an actigraphy device for 2 weeks to analyze the relationship between activity patterns of actigraphy and major components of sleep questionnaires and sleep diary. In each learned group, 3–5 students volunteered to complete the final project using actigraphy recording. All learned groups received de-identified versions of their actigraphy and questionnaire data, which had been processed by a teaching assistant to protect participant confidentiality. To fulfill the project requirement, each learned group could choose to write a report analyzing the sleep performance of their group members and comparing it with the overall class data, or to explore the dataset using any method of their choice.

**Fig. 3:**
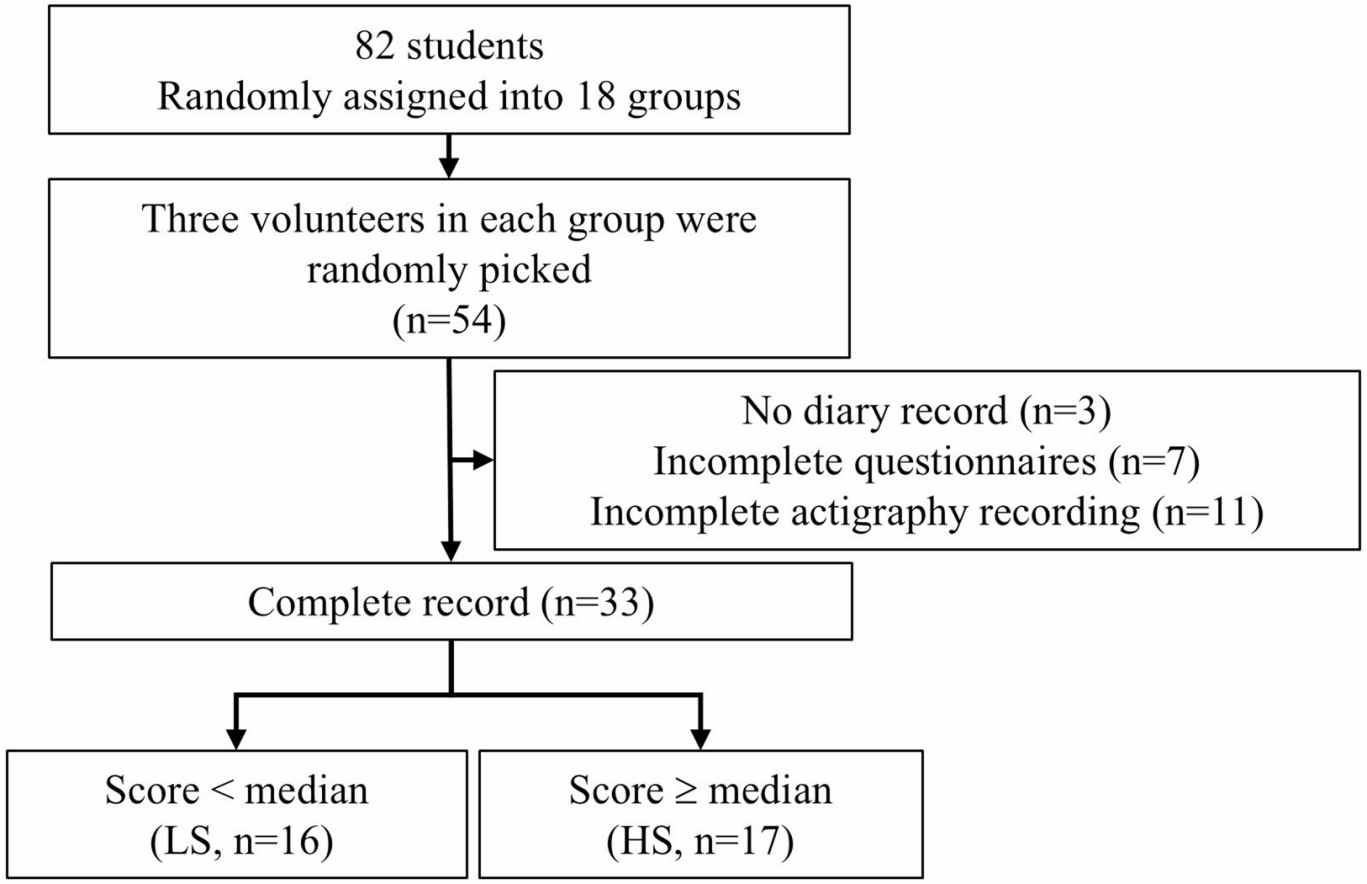
Flow diagram of the student grouping, data collection, and post hoc study design.

The experimental procedure was reviewed and approved by the Human Research Ethics Committee of NCKU (NCKU HREC-E-113–142-2). All procedures were performed in accordance with the Declaration of Helsinki. Informed consent was provided and signed for all participants before the experiment. The inclusion criterion of this study was the participants electing the sleep physiology course. The exclusion criteria were those who had difficulty completing two weeks of recording, and considered at most 5 students in each learned group, due to the limited amount of actigraphy devices. In this study, all volunteers were asked to complete the questionnaires and sleep diaries, and to wear the actigraphy device for two consecutive weeks. After the recording, we identified 3 participants who did not complete the sleep diary, 7 participants who provided incomplete questionnaire data, and 11 participants who failed to complete the actigraphy device recording due to device malfunction or human-related factors (Fig. 3). Thus, the present study included 33 participants with complete data across all measures.

### Experimental procedure

In the current study, we aimed to evaluate the achievement (i.e., score of final exams) with various sleep quality measures, i.e., sleep questionnaires, sleep diaries, and actigraphy. A 14-day consecutive recording period was scheduled, and the final exam was held two weeks after the completion of the recordings. The course took the final exam in the 3/4 semester, not at the end of the semester, to reduce possible interference from other courses. At the beginning of the study, participants provided basic demographic information (e.g., gender, education years, age, height, weight, email) and completed three questionnaires: the Pittsburgh Sleep Quality Index (PSQI), Beck Depression Inventory (BDI), and Perceived Stress Scale (PSS). Participants were then instructed on properly wearing and operating the actigraphy device, including checking battery status and usage precautions. The sleep diary was created using Google Forms and supplemented with a reminder system via the Line messaging application. All participants joined a designated LINE group, through which automated reminders were sent to prompt daily diary completion. During the 14-day monitoring period, participants were asked to wear the actigraphy device continuously, except during activities such as bathing, swimming, or when encountering conditions (e.g., heavy rain) that made wearing the device impractical. The present study particularly paid more attention to complete recordings of nocturnal sleep throughout the two-week monitoring period.

### Questionnaires

The current study used 3 questionnaires. The PSQI was aimed at the assessment of sleep quality. BDI assessed depression symptoms, while the PSS measured participants’ perceived daily stress levels. In addition to the 3 questionnaires, a sleep diary for 14 consecutive days was also performed to evaluate sleep-related components, particularly for on-bed and off-bed times to help clarify actigraphy records.

The Pittsburgh Sleep Quality Index (PSQI), developed by Buysse et al.^56^, is a self-reported questionnaire used to evaluate the quality and pattern of sleep over the past month. This questionnaire consists of nineteen questions, and the resulting score will indicate the extent of seven sleep “components”: sleep quality, sleep onset latency, sleep duration, sleep efficiency, sleep disturbances, usage of sleeping medicine, and daytime dysfunction. A PSQI score of less than 5 is considered a normal sleeper.

The Beck Depression Inventory-Second Edition (BDI-II), formulated by Beck et al.^57^, is a self-reported questionnaire designed to measure the severity of depression. BDI scores are used to classify depression severity into four categories: minimal (0–9), mild (10–18), moderate (19–29), and severe (30–63).

The Perceived Stress Scale (PSS), designed and validated by Cohen et al.^58^, is a self-reported survey used to evaluate psychological stress. The PSS consists of 14 items, including 7 positively worded and 7 negatively worded statements. Each item is evaluated using a 5-point Likert scale. Total scores are used to categorize stress levels as low (0–18), moderate (19–37), or high (38–56).

The sleep diary was administered via Google Forms, allowing participants to enter relevant information, particularly their bedtime and wake time. To enhance compliance, a LINE-based chatbot was developed to send automated reminders, prompting participants to complete their wake-up diary entries each day.

### Actigraphy measure

The actigraphy device contains a three-axis accelerometer (LIS2DH12, STMicroelectronics Inc.), a light sensor (APDS-9300, Broadcom Inc.), and a temperature sensor (Si7050, Silicon Labs) to measure motor activity and environmental situations. All sensor signals are stored in a flash memory with 128 M-bit storage size (W25Q128JVSIQ, Winbond Electronics Corp.) and processed using a microcontroller MDBT42VP512K, which is a system on a chip (SoC) solution for Nordic nRF52832 (Nordic Semiconductor Inc.). A micro-USB module is embedded for data transmission with computers for data downloading and further analysis. The actigraphy device relies on a lithium battery of 3.7 V/140mAh to sustain continuous recording for 30 days. The circuit board size is 17 × 27 × 6 mm^3^ and the weight of the actigraphy device is 25.8 g. Participants were instructed to wear the actigraphy device on their non-dominant hand.

For the data recording, the three-axis acceleration signals (unit: mg) were recorded with a 25 Hz sampling rate, and the sampled signals of 1 s were averaged to reduce data size and signal fluctuation^37^. Temperature (unit: °C), and illumination (unit: Lux) were recorded with a sampling rate of 1 Hz. The information on the recording date and time was also noted.

### Actigraphy data processing

For the process and analysis of the recorded actigraphy data, a graphical user interface (GUI) was developed with the MATLAB environment and can be used to perform bedtime/wake time labeling, sleep-wake identification, and sleep parameter calculation.

#### Bedtime/wake time labeling

The present study first referred to the subjective information of the sleep diary (if available) to estimate events of daily nocturnal sleep. Then, we subsequently referred to the objective measures of temperature (> 30 °C) and illumination (< 10 lx) to more precisely determine the bedtime (time for going to sleep). Moreover, we also referred to the z-axis signal of the accelerometer with a < 50 mg fluctuation to confirm the bedtime. The criteria for the objective waking time determination and the requirement were z-axis fluctuation > 1 g and illumination > 400 lx.

#### Sleep-wake-staging

After determining the subject’s bedtime and wake time, the data in bed were segmented into 30-second segments (epochs) and each epoch was identified as either sleep or wakefulness according to the sleep-wake staging algorithm proposed in^37^. For each epoch, the accelerometer signal peaks exceeding a predefined threshold were identified as activity peaks, and the shortest time between two successive activity peaks was defined as a feature called the peak-to-peak interval (PP interval). If more than three activity peaks were detected and the minimum PP interval in an epoch was low (< 11 s), the epoch was considered to contain evident movement and was labeled as an activity epoch. The movement density (MD) of an epoch was determined by the number of activity epochs in the window of that epoch and the neighboring epochs (25 epoches). An epoch with a larger MD will more likely be identified as wakefulness.

#### Sleep parameters calculation

This study selected 4 primarily clinical sleep parameters, e.g., SE, SOL, WASO, and TST. These indices are associated with different aspects of sleep disturbances: poor sleep quality is reflected by SE, difficulty initiating sleep by SOL, difficulty maintaining sleep by WASO, and reduced sleep duration by TST. These are also the major sleep metrics commonly derived from actigraphy^37^. Specifically, TST represents the entire length of epochs that are classed as “sleep.” SOL is the time interval between bedtime and the occurrence of the first sleep epoch. WASO represented the cumulative duration of epochs labeled as “wake” occurring after sleep onset. SE was calculated as the percentage of TST divided by time in bed, defined as the interval between bedtime and wake time.

In addition to the daily sleep parameters, this study also examined variability across days. For each participant, the MAD of the sleep parameters was calculated over the entire recording period, as well as separately for weekdays and weekends. The MAD was calculated as below.

1 $$\:Mean\:absolute\:deviation\:\left(MAD\right)=\:\frac{\sum\:\left|{x}_{i}-\stackrel{-}{x}\right|}{n}$$

Where *n* refers to the number of data points and x represents the value of the sleep parameters. Compared with traditional standard deviation measures^30^, the MAD is more tolerant of extreme values (outliers) and produces a higher effect size on statistical assessment^44^. The terms “weekday” and “weekend” are defined according to the prior sleep research^59,60^. The evenings before a non-working day are weekend nights, specifically Friday and Saturday nights. In contrast, nights from Sunday to Thursday are categorized as weekday nights.

### Statistical analysis

All statistical analyses were conducted using SigmaPlot 14.0. A p-value < 0.05 was considered statistically significant. This study calculated effect size of all sleep metrics. In the current study, we classified the participants into two groups based on their final exam score: low score (LS, score < median) and high score (HS, score≥median). The normality test (Shapiro-Wilk test) and equal variance test (Brown-Forsythe test) were assessed for the two groups’ data before using the parametric statistical analysis. Body mass index (BMI) was calculated as weight (kg) divided by height squared (m^2^). For descriptive statistics, demographic variables (age, years of education, BMI) and questionnaire scores (PSQI, BDI, PSS) were compared between groups using Student’s t test when assumptions were met, or the Mann–Whitney U test otherwise. Fisher’s exact test was applied to assess gender distribution between groups. Group comparisons of sleep parameters (TST, SOL, WASO, SE), their corresponding MADs, and final exam scores were analyzed using Student’s t-test or the Mann–Whitney U test, as appropriate. A mixed-model analysis of variance (ANOVA) was employed to evaluate the effects of group and time (weekday vs. weekend) on sleep parameters and their MADs. An analysis of covariance (ANCOVA) was employed if demographic variables showed significant differences between the two groups. Pearson correlation analysis was used to examine correlations between all sleep parameters (and their MADs) and the final exam score.

## Supplementary Information

Below is the link to the electronic supplementary material.

Supplementary Material 1

## Data Availability

The datasets used and/or analysed during the current study available from the corresponding author on reasonable request.
