# Supplementary material for: Better sleep is associated with higher academic performance from an actigraphy-based analysis of sleep consistency and grades in college students
Source: Sci Rep. 2025 Dec 26;16:3766. doi: 10.1038/s41598-025-33775-0 (PMC12852834; doi:10.1038/s41598-025-33775-0)
Supplement: Supplementary file 1 — Supplementary Material 1 [file 41598_2025_33775_MOESM1_ESM.docx]

In this study, no statistically significant difference was found in gender distribution between the two groups (Table 1), although the *p-value* for gender was 0.057, which approaches the conventional threshold for significance. To account for gender as a potential confounding factor, we conducted an ANCOVA analysis. The results of these additional analyses are summarized in Tables S1 and S2. Table S1 shows that the four objective sleep parameters maintained similar significant patterns, with the exception of the MAD of SOL, which did not reach statistical significance. Table S2 also reflects consistent significant patterns in the four sleep parameters, except for WASO and the MAD of SE, which did not show significant differences. Notably, the p-values for these non-significant items remained close to 0.1. Overall, the majority of the statistical results continue to support our main findings regarding the significant correlation between sleep quality and academic performance.

Table S1. Comparison of statistical results by the ANCOVA test (considering the gender as confounding factor) and the Student t test of this study.

| Parameter | *p*-value (ANCOVA by the F-test) | | *p*-value (T-test) |
| --- | --- | --- | --- |
|  | Gender | Group |  |
| SE | 0.422 | **<0.001** | **<0.001** |
| SOL | **0.019** | **0.009** | **<0.001** |
| WASO | 0.757 | 0.097 | 0.177 |
| TST | 0.757 | 0.400 | 0.301 |
| MAD |  |  |  |
| SE | 0.690 | **0.003** | **<0.001** |
| SOL | 0.072 | 0.130 | **0.020** |
| WASO | 0.433 | 0.417 | 0.228 |
| TST | 0.808 | **0.013** | **0.010** |

Table S2. Comparison of statistical results by the ANCOVA test (considering the gender as confounding factor) and the ANOVA test of this study.

| Parameter | *p*-value (ANCOVA test) | | *p*-value of Group (ANOVA test) |
| --- | --- | --- | --- |
|  | Gender | Group |  |
| SE | 0.073 | **0.019** | **<0.001** |
| SOL | 0.037 | **0.013** | **<0.001** |
| WASO | 0.875 | 0.115 | **0.028** |
| TST | 0.700 | 0.753 | 0.528 |
| MAD |  |  |  |
| SE | 0.500 | 0.085 | **0.010** |
| SOL | 0.136 | **0.016** | **0.001** |
| WASO | 0.562 | 0.372 | 0.186 |
| TST | 0.547 | **0.025** | **0.012** |
